# Greener Schoolyards, Greener Futures? Greener Schoolyards Buffer Decreased Contact With Nature and Are Linked to Connectedness to Nature

**DOI:** 10.3389/fpsyg.2020.567882

**Published:** 2020-11-13

**Authors:** Sílvia Luís, Ronisa Dias, Maria Luísa Lima

**Affiliations:** ^1^Centro de Administração e Políticas Públicas (CAPP-ISCSP), Instituto Superior de Ciências Sociais e Políticas (ISCSP), Universidade de Lisboa, Lisbon, Portugal; ^2^Centro de Investigação e de Intervenção Social (CIS-IUL), Lisbon, Portugal; ^3^Iscte-Instituto Universitário de Lisboa (Iscte), Lisbon, Portugal

**Keywords:** schoolyard, contact with nature, attitudes towards nature, social competences, connectedness to nature, perceived restorativeness

## Abstract

Children are spending less time in nature. They are not taking advantage of the benefits that are related to experiencing nature, such as the improvement of attentional capacity and stress reduction. Furthermore, they are also losing the opportunity to assess nature in a more positive way and to become more connected to nature, factors that appear to be fundamental to securing a greener future. To overcome this problem, researchers have been focusing on increasing children’s contact with nature in schools and in promoting garden-based learning programs. Children spend most of their time in school, where they face many cognitive and relational demands. As such, schools might be the ideal context to increase children’s contact with nature with more empirical research being needed to understand the effects that greener schools might have. The goal of this study is to explore the effects of schoolyards in children’s perceived restorativeness experiences, attitudes towards nature, connection to nature, and social competences. For that, we studied children (*N* = 132) from three elementary schools with different schoolyards: a school with cement yard with a few trees, a school with green areas, and a school where many parts of the yard were earthen and there was a vegetable garden that the students could cultivate as part of an ongoing garden-based learning program. The results of a questionnaire confirmed that greener schoolyards were related to stronger restorative experiences. As such, children might benefit from improving their attentional capacity during breaks. Unexpectedly, the perceived restorativeness effect was stronger for children who usually had lesser contact with nature than for children who contacted more with nature. This suggests that having schools with green yards might buffer some of the effects of reduced contacts with nature outside of school. The effects of the schoolyard in children’s social competences did not appear to emerge. However, children that attended the school with the greener schoolyard had more positive attitudes and were more connected to nature than children from the other two schools. This further suggests that designing greener schoolyards might be an opportunity to contribute to reversing global environmental challenges.

## Introduction

Today’s children spend less time playing outdoors and exploring nature than did previous generations, as a result of both urbanization and the overscheduling and micromanagement of children’s lives (Clements, 2004; Soga and Gaston, 2016). Pyle (1993) has termed this alienation from nature as *extinction of experience* back in 1993. Later, Louv (2005) illustrated the severity of this phenomenon that has kept on increasing using the term *nature-deficit disorder*. Indeed, studies show that the loss of interaction with nature diminishes a wide range of benefits relating to general well-being and health (Hartig et al., 2014; Collado and Corraliza, 2016), as well as to improvement of attentional capacity and stress reduction (Ulrich, 1983; Kaplan, 1995). Furthermore, it also discourages positive attitudes (Zhang et al., 2014), connectedness to nature, and sustainable behaviors (Barrera-Hernández et al., 2020). As such, it can be an obstacle to reversing global environmental challenges. Recent research has suggested that one way to bypass this problem is to increase children’s contact with nature in schools, as this is where children spend most part of their time (Collado and Corraliza, 2016). In this vein, the “Child Friendly Cities Initiative,” a UNICEF-led initiative launched in 1996, has defined that an important characteristic of communities is children’s access to green spaces, and schools may play an important role in promoting it. Research has focused the potentialities of designing greener schools and, more recently, in implementing garden-based learning programs in schools. However, more empirical research is needed to legitimize the need for greener schools (Eugenio-Gozalbo et al., 2020).

The goal of this study is to contribute by exploring and comparing the effects of schoolyards in three schools where the presence of green (i.e., natural) elements varies. We focus on a well-known immediate effect, the restorative experience of the schoolyard, and on longer term effects, attitudes and connectedness to nature and social competences.

Since Ulrich’s (1983) and Kaplan’s (1995) seminal works on the effects of nature on restoring directed attention and stress recovery, many studies have been illustrating the benefits of being in green spaces (e.g., Keniger et al., 2013). The attention restoration theory, in particular, states that directed attention is used when a certain object or task does not attract out attention voluntarily and attentional effort is required (Kaplan, 1995). Directed attention plays a central role in achieving focus and controls distraction through the use of inhibition. Any prolonged mental effort leads to directed attention fatigue, which is accompanied by negative feelings, such as irritability. The best way to recover from fatigue is by spending time in what was defined as restorative environments (Kaplan and Kaplan, 1989). Restorative environments have four characteristics: fascination, the capacity to effortlessly capture people’s attention; being-away, the extent to which the environment enables people to get away from their daily routine; extension, referring to an environment that is rich and encourages exploitation; and compatibility, the extent of the fit between the individuals’ interests and that environment. Posterior studies have further differentiated between being-away physically and psychologically (e.g., Laumann et al., 2001). A substantial body of research evidences that natural environments are more restorative than built ones, indicating the importance of contacting with nature (Berto, 2005; Corraliza et al., 2012; Li and Sullivan, 2016; Negrín et al., 2017). Children at school need direct attention to deal with cognitive tasks and relational situations. As such, it is of paramount importance to illustrate how the presence of green elements in schoolyards turns them into a more restorative environment that might facilitate children’s recovery of direct attention.

Corraliza et al. (2012) studied the possible difference in terms of the perceived restorativeness of school playgrounds depending on the level of nature they contained. Their results already support that it is important to include nature in school playgrounds, since in playgrounds with a greater number of natural elements children have higher perceived restoration. Furthermore, Kelz et al. (2015) illustrated that after the redesign (greening) of a schoolyard, students perceived the environment as more restorative, had diminished physiological stress levels, and enhanced their psychological well-being. In this study, we aim to corroborate the restorative effect of green schoolyards and to ascertain if it might be facilitated for children that have frequent contact with nature. There is evidence suggesting that adults who contact more with nature during their leisure time also contact more with nature during their working time (e.g., take a daily park walk during their lunch break) and have higher vigor, dedication, and absorption at work (Hyvönen et al., 2018). In this vein, we explored if restorativeness was higher for children who had frequent contact with nature, as regular contact with nature might lead children to actively seek the green elements in schoolyards thereby facilitating the triggering of restoration processes. Also, when children contact with nature more frequently, they might become more competent in restoring their attentional capacity. This would suggest that there could be room for training children to improve their competence in restoring their attentional resources.

Besides the immediate effects that greener schoolyards might have on children, we also explored effects on children’s attitudes and connectedness to nature and on social competences.

Attitudes towards nature refer to a general evaluative reaction to nature. Attitudes can change easily following intervention experiences, particularly when they are not important to people or influential on action (Howe and Krosnick, 2017). As such, in addition to attitudes, we focused on connectedness to nature which is a broader and trait-type concept. Changes in connectedness to nature should take place over a relatively long period of time, rather than as the result of a single experience. Connectedness to nature refers to a stable state of consciousness comprising interdependent cognitive, affective, and experiential traits that reflect a sustained awareness of the interrelatedness between one’s self and nature (Zylstra et al., 2014). Barrera-Hernández et al. (2020) have recently shown that children who are more connected to nature carry out more sustainable behaviors.

School design might also influence social interaction between children (Olivos, 2010). Lastly, we explored if greener schoolyards were related to the development of social competences, as the restoration of directed attention should lead to a more positive emotional state and relate with the ability to reflect and have appropriate social behavior (Kaplan, 1995). Social competence plays a key role in children’s adaptation to school functioning, influencing relations with teachers, peer acceptance, and academic achievement. Raimundo et al. (2012) indicate that children’s early life experiences and parental modeling of emotional expression are crucial predictors of social competences but that it is also possible to promote social competence in children via intervention programs implemented inside or outside the school. Carrus et al. (2015) assessed children’s performance in structured activities and behavior in free play through systematic observation, after time spent in outdoor green vs. indoor space of the same school. Their findings suggest that contact with outdoor green spaces positively influenced children’s social behavior. A recent systematic review also illustrates that exposure to green space may potentially increase prosocial behavior among children and adolescents, with some contingencies, e.g., child’s sex and ethnic background (Putra et al., 2020). However, it is highlighted that the volume and quality of this evidence is not yet enough to draw conclusions on causality.

In sum, the goal of this study was to investigate if children had more restorative experiences in greener schoolyards, as well as more positive attitudes and higher connection to nature, and higher social competences. To achieve these goals, we conducted surveys by questionnaire in selected schools with different yards in the city of Lisbon, Portugal: a school with cement yard with a few trees, a school also with cement yard but with many green areas (trees, grass, and beds), and a school where many parts of the yard were earthen and there was a vegetable garden that the students could cultivate as part of an ongoing garden-based learning program.

## Materials and Methods

### Schools and Participants

**FIGURE 1:**
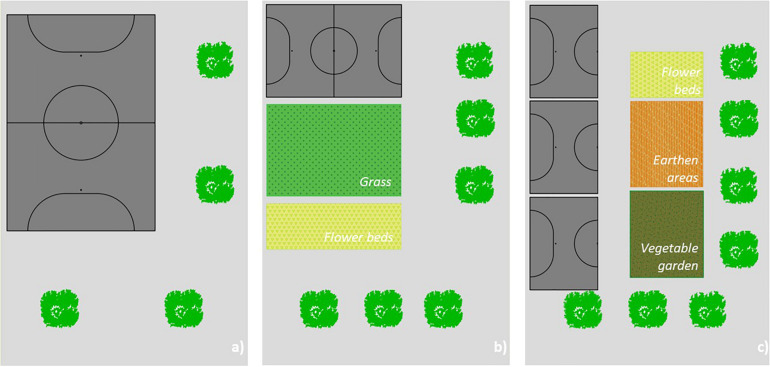
Conceptual representation of the schoolyards: **(a)** cement yard; **(b)** green areas; **(c)** earthen areas and vegetable garden.

A cross-sectional research design was used to study children from schools with different yards. Schools that entered our schoolyard type classification in the Lisbon urban area were contacted to participate in the study. Three schools that meet our criteria agreed participating. The school with the *cement yard* had ample outdoor space with some trees on the perimeter and a playing field. Outside the schoolyard, there was a space with a sand box that has previously been a vegetable garden but was not cared for. After this study was completed, the vegetable garden was rehabilitated. The schoolyard with *green areas* had beds and a green area with paintings made by children on nature, two roofs, and a playing field. The schoolyard with *earthen areas and vegetable garden* had three playing fields, cemented areas and beds, earthen areas where children can picnic, two roofs, and a well-attended vegetable garden. The conceptual representation of these elements is schematized in Figure 1.

After we gathered authorizations from the schools and parental consent, children from elementary teaching (*N* = 132) were asked to respond to the survey. Fifty-five percent of the children were female, and their mean age was 10 years old (*M* = 9.77, *SD* = 0.99; age ranged between 8 and 14 years old). The three schools were public. The sociodemographic data for each school is presented in Table 1.

**TABLE 1:** Sociodemographic data by school.

**Schoolyard**	**Cement**	**Green areas**	**Earthen areas, vegetable garden**
*N*	37	46	49
Age, mean (SD)	9.76 (0.80)	10.09 (1.31)	9.47 (0.62)
Sex (% female)	45.9%	63.0%	53.1%

Children completed the survey after their afternoon breaks in their classrooms. An interviewer read all questions and was available to respond to doubts. Children spent between 20 and 30 min to complete the survey. At the end, the interviewer assigned a code number to each survey that was paired with the children’s name in a database to guarantee anonymity.

The social competences of the children were assessed by their teachers (two teachers in the *cement yard* school, three teachers in the *green areas* yard school, and three teachers in the *earthen areas and vegetable garden* yard school). The interviewer instructed the teachers on how to complete the surveys and assigned each children’s code number.

### Measures

All variables were gauged using a five-point Likert-type scale. Children responded to the survey face to face. Their survey included measures of restorativeness, attitudes towards nature, connectedness to nature, and frequency of contact with nature that were translated to Portuguese. Teachers were asked to evaluate the social competences of their students.

#### Restorativeness

Perceived restorativeness was measured using the Perceived Restorative Components Scale for Children II (Bagot et al., 2007; Corraliza et al., 2012). The scale is made up of 14 items and 5 factors: fascination (e.g., “There are lots of interesting places in the schoolyard”), being away physically (e.g., “Being in the schoolyard, I feel as though I am in different surroundings than when I am in the classroom”), being away psychologically (e.g., “When I’m in the schoolyard I feel free from schoolwork and class time”), compatibility (e.g., “The things I like to do can be done in the schoolyard”), and stimulatory diversity (e.g., “I can do all the things that can be done in the schoolyard”). The measure presented an adequate level of internal consistency in our sample (α = 0.79).

#### Attitudes Towards Nature

To measure attitudes towards nature, we used the children’s environmental perceptions scale (Larson et al., 2011; Collado et al., 2015). This scale is comprised by 15 items and measures two distinct components of environmental orientation: eco-affinity and eco-awareness. Eco-affinity refers to the personal interest in nature and intentions to engage in proenvironmental behavior (e.g., “I am interested in learning new ways to help protect plants and animals”). Eco-awareness refers to cognitive evaluations of environmental issues and sustainability of (e.g., “People need plants to live”). Internal consistency was acceptable (α = 0.68).

#### Connectedness to Nature

The Connection to Nature Index is a questionnaire developed by Cheng and Monroe (2012). It consists of 16 items divided by four subscales: enjoyment of nature (e.g., “I like to see wildflowers in nature”), empathy for creatures (e.g., “I feel sad when wild animals are hurt”), sense of oneness (e.g., “Humans are part of the natural world”), and sense of responsibility (e.g., “My actions will make the natural world different”). Internal consistency was acceptable (α = 0.70).

#### Contact With Nature

Children’s self-reported contact with nature was measured using six items adapted from previous research (Gotch and Hall, 2004; Collado et al., 2015). Children were asked four questions about how many times they had conducted activities in the last 12 months, on a scale from 1, *never*, to 5, *more than 10 times* (e.g., “Spend time in natural places?”), and two questions about their daily and weekend experience in nature, on a scale from 1, never, to 5, always (“Do you play in natural places after school time?”). Internal consistency was acceptable (α = 0.66).

#### Social Competences

We used the Portuguese adapted and reduced version (Raimundo et al., 2012) of the social competence scale from the School Social Behavior Scales (Merrell, 2002). It is a 10-item rating instrument of children and adolescent’s behavior to be used by teachers and other school personnel, on a scale from 1, *never*, to 5, *very often*. This scale includes three subscales: self-management/compliance (e.g., “Behaves properly at school”), peer relations (e.g., “Cooperates with the other students”), and academic behavior (e.g., “Listen to and carry out the instructions provided by teachers”). Internal consistency was high (α = 0.94).

## Data Analysis and Results

### Restorativeness Effects of Yards Moderated by Children’s Contact With Nature

First, we tested if children’s perceived restorativeness was higher while at greener schoolyards, particularly when they had a greater contact with nature. Means and standard deviations for perceived restorativeness in the three schoolyards are presented in Table 2.

**TABLE 2:** Descriptive statistics and ANOVA results.

**Schoolyard**	**Perceived restorativeness**		**Attitudes towards nature**		**Connection to nature**		**Social competences**	
	
	***M* (SD)**							
Cement	3.66 (0.61)^*a*^		4.36 (0.57)^*a*^		4.09 (0.44)^*a*^		3.67 (0.88)^*a*^	
Green areas	4.21 (0.53)^*b*^		4.21 (0.71)^*a*^		4.14 (0.48)^*a*^		3.09 (0.73)^*b*^	
Earthen areas, vegetable garden	4.32 (0.40)^*b*^		4.70 (0.31)^*b*^		4.39 (0.34)^*b*^		3.66 (0.87)^*a*^	
	*F*(2, 129)	η*_*p*_*^2^	*F*(2, 129)	η*_*p*_*^2^	*F*(2, 129)	η*_*p*_*^2^	*F*(2, 125)	η*_*p*_*^2^
	18.58***	0.224	10.19***	0.136	6.09**	0.086	7.01**	0.101

Measures vary between 1 and 5. Means with different superscript letters, within the same column, are significantly different from each other (Bonferroni’s test, p < 0.050). **p < 0.010, ***p < 0.001.

The schoolyards’ perceived restorativeness was medium/high. In vein with our expectation, the schoolyards with greener areas scored higher in the perceived restorativeness components. One-way ANOVA results support the existence of significant differences between schoolyards (see Table 2). Bonferroni test indicates that in the school with cement yard, children’s perceived restorativeness was (a) lower than in the school with green areas in the yard, 95% CI [−0.82, −0.27], and (b) lower than children’s perceived restorativeness in the school with earthen parts of the yard and with the vegetable garden, 95% CI [−0.93, −0.38].

**FIGURE 2:**
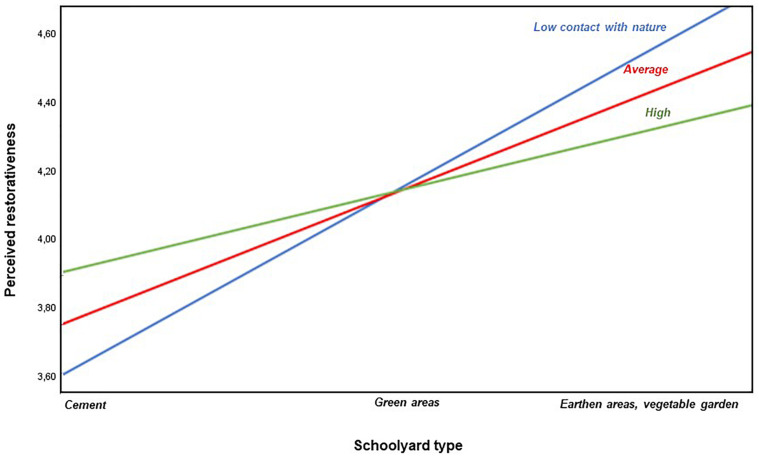
Moderation effect of children’s contact with nature on the relation between schoolyard type and perceived restorativeness (values are non-centered for better visualization).

Children’s frequency of contact with nature was, in average, medium (*M* = 3.50, *SD* = 0.80). To test if this effect was moderated by the children’s contact with nature, we used the PROCESS macro for SPSS version 3 (Hayes, 2018), which is based on ordinary least square regression and path analysis. For moderation analyses, variables were centered except when stated otherwise. The number of bootstrap samples for percentile bootstrap confidence intervals was 5,000. The moderation model was first tested controlling for sex and age. These variables did not have a significant effect and were removed to preserve a maximum degree of freedom. A model entering the type of schoolyard as predictor and contact with nature as moderator explained 23% of the variability of perceived restorativeness, *F*(3, 128) = 12.48; *p* < 0.001. As expected from the ANOVA results, the relation between schoolyards and perceived restorativeness was positive, *b* = 0.35, *t* = 5.84, *p* < 0.001, 95% CI [0.23, 0.46]. However, the moderation effect was negative, *b* = −0.17, *t* = −2.37, *p* = 0.019, 95% CI [−0.31, −0.03], meaning that decreases in contact with nature actually increased the positive effect of greener schoolyards on the perceived restorativeness. Simple slope analyses show that the effect was stronger when contact with nature was one standard deviation below average, *b* = 0.48, *t* = 5.38, *p* < 0.001, 95% CI [0.30, 0.66], being lower when it was one standard deviation above average, *b* = 0.21, *t* = 2.88, *p* = 0.005, 95% CI [0.07, 0.36], as illustrated in Figure 2. The Johnson-Neyman technique, which algebraically derives the moderator values where the effects are significant (see Hayes, 2018), indicated that moderation effects occurred until a very high value of contact with nature, 4.56, *b* = 0.17, *t* = 1.98, *p* = 0.050, 95% CI [0.00, 0.34], non-centered values.

### Attitudes Towards Nature, Connectedness to Nature, and Social Competences in the Different Schoolyards

Means and standard deviations are presented in Table 2. As expected, children attending the school with the yard with earthen areas and the vegetable garden had more positive attitudes towards nature and were more connected to nature. One-way ANOVA results supports the existence of significant differences between schoolyards (see Table 2). Bonferroni test indicates that in the school with the yard with earthen areas and the vegetable garden, children’s attitudes towards nature were (a) higher than in the school with the cement yard, 95% CI [0.05, 0.63], and (b) higher than in the school with the yard with green areas, 95% CI [0.22, 0.77]. Likewise, in the school with the yard with earthen areas and the vegetable garden, children were (a) more connected to nature than children in the school with the cement yard, 95% CI [0.10, 0.47], and (b) more connected than children in the school with the yard with green areas, 95% CI [0.07, 0.41].

Significant differences in the children’s social competences between schoolyards were also found (see Table 2). However, differences were not in line with our expectations. Bonferroni test indicates that in the school with the yard with green areas, children not only had (a) lower social competences than children in the school with earthen areas and the vegetable garden, 95% CI [−0.91, −0.24], but also (b) lower social competences than children in the school with the cement yard, 95% CI [−0.95, −0.20].

We further analyzed if the results endured when taking into account the effects of sex and age. To simplify, we ran correlation analyses controlling for these variables. Results were sustained. The correlation between schoolyards and the study variables are significantly positive for all variables except social competences: *r*(124)_*attitudes*_ = 0.25, *p* = 0.004, 95% CI [0.08, 0.41]; *r*(124)_*connectedness*_ = 0.29, *p* = 0.001, 95% CI [0.12, 0.44]; *r*(124)_*social*_*_competences_* = −0.01, *p* = 0.875, 95% CI [−0.19, 0.16].

## Discussion

This study provides empirical evidence on the benefits of designing greener schools and suggests that green schoolyards can contribute to mitigate the *nature deficit disorder*. We found that greener schoolyards promoted stronger restorative experiences in elementary school children, who have long periods of learning and, therefore, should benefit from directed attention restoration during school breaks. This result is in line with previous studies that show the restorative effects of green schoolyards on students (Corraliza et al., 2012; Kelz et al., 2015). Of importance, the perceived restorativeness effect was stronger for children who had lesser contact with nature. Although unexpected, this a positive effect. It suggests that having schools with green yards might buffer the effects of reduced contacts with nature outside of school. This finding is a starting point for future studies to comprehend the protective effects that green schoolyards might have.

Children that attended the schools with greener schoolyards also had more positive attitudes and were more connected to nature. This suggests that designing green schoolyards might have effects on both volatile (attitudes) and stable-type (connectedness to nature) variables, being an opportunity to contribute to reversing global environmental challenges. As Barrera-Hernández et al. (2020) illustrate, children connected to nature behave in a sustainable manner.

It should also be highlighted that there were no differences in the perceived restorativeness of the schoolyard with green areas and the schoolyard with earthen areas and the vegetable garden. However, when it came to attitudes and connection to nature, the schoolyard with earthen areas and the vegetable garden appears to have a greater influence. The importance of simultaneously greening and increasing biodiversity exposure has also been illustrated by Puhakka et al. (2019) in six urban daycare yards in Finland. Following the yards’ transformation, daycare personnel and parents report that 3–5-years-old children became more engaged and connected to nature and also increased their physical activity and perceived well-being.

We did not find the pattern of results that we expected for social competences. As this is a correlational study, we can only speculate why this effect did not emerge. It might be related to the characteristics of the instrument that was used to assess social competences. The School Social Behavior Scales rely on the assessment of children by teachers and, therefore, might be more vulnerable to judgmental bias than other types of instruments, which might have influenced the results. Another possibility is that the effects of outdoor green spaces are contingent to the activities performed immediately after the restorative experience. In the study done by Carrus et al. (2015), children’s performance in structured activities and behavior in free play were assessed through systematic observation, after time spent in outdoor green vs. indoor space, and suggest that contact with outdoor green spaces positively influences children’s social behavior in a subsequent task. In addition, it has been observed that social interaction in schools depends on the interplay between many variables, such as the number of children per class or personality traits (e.g., Olivos et al., forthcoming). As such, additional studies are required to explain this result.

A study limitation that needs to be acknowledged is that the schoolyards that we studied had different characteristics besides the presence of natural elements that might have influenced the results and are beyond our control. Of relevance, the school with earthen areas had a curriculum that included a garden-based learning program. An avenue for future studies would be to unravel the effects that school’s design and curriculum might have. Despite that, this study contributes to legitimize the key role that green schoolyards have in promoting restorative experiences and enhancing attitudes and connection to nature which might be vital for securing greener futures. City planners and policy makers need to focus more attention and effort on planning how best to (re)connect children with nature, which should contribute greatly both to achieving healthy societies and overcoming a wide range of environmental issues.

## Data Availability Statement

The raw data supporting the conclusions of this article will be made available by the authors, without undue reservation.

## Ethics Statement

Ethical review and approval was not required for the study on human participants in accordance with the local legislation and institutional requirements. Written informed consent to participate in this study was provided by the participants’ legal guardian/next of kin.

## Author Contributions

SL was involved in the conceptualization, data analysis, and writing of the manuscript. RD was involved in the investigation, data collection, and editing and reviewing of the manuscript. ML was involved in the conceptualization and methodology. All authors contributed to the article and approved the submitted version.

## Conflict of Interest

The authors declare that the research was conducted in the absence of any commercial or financial relationships that could be construed as a potential conflict of interest.
